# Prevalence of Dementia and Cognitive Impairment in East Africa Region: A Scoping Review of Population-Based Studies and Call for Further Research

**DOI:** 10.3233/JAD-240381

**Published:** 2024-08-13

**Authors:** Muluken A. Yenesew, Janina Krell-Roesch, Betelhem Fekadu, Dabere Nigatu, Aklilu Endalamaw, Alemtsehay Mekonnen, Mulugeta Biyadgie, Gizachew Y. Wubetu, Alemu T. Debiso, Kassu M. Beyene, Teshome S. Kelkile, Daniel A. Enquobahrie, Tesfaye B. Mersha, Danielle E. Eagan, Yonas E. Geda

**Affiliations:** a School of Public Health, College of Medicine and Health Sciences, Bahir Dar University, Bahir Dar, Ethiopia; b Institute of Sports and Sports Science, Karlsruhe Institute of Technology, Karlsruhe, Germany; c Department of Psychiatry, College of Medicine and Health Sciences, Wollo University, Dessie, Ethiopia; d School of Public Health, University of Queensland, Brisbane, Australia; e School of Health Sciences, College of Medicine and Health Sciences, Bahir Dar University, Bahir Dar, Ethiopia; f School of Medicine, College of Medicine and Health Sciences, Bahir Dar University, Bahir Dar, Ethiopia; g Amhara Public Health Institute, Amhara Region, Bahir Dar, Ethiopia; h College of Medicine and Health Sciences, Hawassa University, Hawassa, Ethiopia; i Department of Neurology, Barrow Neurological Institute, Phoenix, AZ, USA; j Horizon Health Network, New Brunswick, Canada; k Department of Epidemiology, School of Public Health, University of Washington, Seattle, WA, USA; l Cincinnati Children’s Hospital Medical Center, University of Cincinnati, Cincinnati, OH, USA; m Department of Neuropsychology, Barrow Neurological Institute, Phoenix, AZ, USA; n Department of Neurology and the Franke Barrow Global Neuroscience Education Center, Barrow Neurological Institute, Phoenix, AZ, USA

**Keywords:** Alzheimer’s disease, cognitive impairment, dementia, East Africa, prevalence, scoping review

## Abstract

**Background:**

Population-based research on the prevalence and determinants of dementia, Alzheimer’s disease, and cognitive impairment is scarce in East Africa.

**Objective:**

To provide an overview of community- and population-based studies among older adults on the prevalence of dementia and cognitive impairment in East Africa, and identify research gaps.

**Methods:**

We carried out a literature search using three electronic databases (PubMed, Scopus, Google Scholar) using pertinent search terms.

**Results:**

After screening 445 publications, we identified four publications on the population-based prevalence of dementia, and three on cognitive impairment. Prevalence rates varied from 6– 23% for dementia, and 7– 44% for cognitive impairment, among participants aged≥50–70 years. Old age and a lower education level were risk factors for dementia and cognitive impairment. Physical inactivity, lack of a ventilated kitchen, and history of central nervous system infections and chronic headache were associated with increased odds of dementia. Female sex, depression, having no spouse, increased lifetime alcohol consumption, low income, rural residence, and low family support were associated with increased odds of cognitive impairment. Potential misclassification and non-standardized data collection methods are research gaps that should be addressed in future studies.

**Conclusions:**

Establishing collaborative networks and partnering with international research institutions may enhance the capacity for conducting population-based studies on dementia and cognitive impairment in East Africa. Longitudinal studies may provide valuable insights on incidence, as well as potential risk and protective factors of dementia and cognitive impairment, and may inform the development of targeted interventions including preventive strategies in the region.

## INTRODUCTION

Dementia is characterized by impairment in one or more cognitive domains that lead to functional decline.^1^ Dementia can have various causes, with the most common being Alzheimer’s disease.^2^Alzheimer’s disease is a neurodegenerative disease characterized by neuritic plaques, neurofibrillary tangles, and neuronal loss^2,3^ Cognitive impairment is an umbrella term that refers to an impairment in cognition such as memory, attention or executive function, language, and visuospatial skills.^4^For the purpose of this review, we mainly focused on mild cognitive impairment, which is the intermediate stage between normal cognitive aging and dementia, and is considered a high-risk state for progression to dementia. In contrast to dementia, persons with mild cognitive impairment have essentially normal functional activities.^5^

Dementia and cognitive impairment present significant public health challenges globally, particularly in aging populations,^6,7^ and can pose significant economic and caregiving burden on societies and families alike, particularly in low- and middle-income countries and regions.^8^ The burden of dementia is rising in Africa.^9,10^ In light of demographic changes and aging societies across the globe, by the end of 2050, the projected number of persons living with dementia in the eastern part of sub-Saharan Africa will increase by 357% [estimated range: 323%–395%], and this estimate is one of the highest compared to other low- and high-income countries.^11^However, to date, no nation in sub-Saharan Africa has developed a stand-alone or integrated national dementia strategic plan to direct the effort to improve dementia care.^12^

As populations continue to age and life expectancy increases, a better understanding of the prevalence of dementia and cognitive impairment is crucial for effective healthcare planning, resource allocation, and development of targeted interventions.^11^This is especially important in East Africa region, which has the highest number of older population compared to other regions of Africa.^9^ East Africa encompasses a number of countries including Burundi, Comoros, Djibouti, Ethiopia, Eritrea, Kenya, Rwanda, Seychelles, Somalia, South Sudan, Sudan, Tanzania, and Uganda.^13^ While East African countries share similarities in terms of sociodemographic and socioeconomic characteristics and level of healthcare systems,^13^ they also exhibit significant variations in lifestyle, cultural, and other factors that may be related to dementia and cognitive impairment.

Old age, lower education levels, limited access to healthcare services, and the presence of comorbid medical conditions such as hypertension and diabetes are associated with an increased risk of dementia and cognitive impairment.^9,10,14–19^ Cultural beliefs, stigma, and lack of awareness about dementia and cognitive impairment may also affect early detection and diagnosis, potentially leading to an underestimation of prevalence rates.^20,21^

Previous reviews conducted in sub-Saharan Africa showed that the reported prevalence of dementia varied substantially (range:<1%–21.6%).^9,22–25^ However, those reported prevalence rates may not accurately reflect the true prevalence of dementia in sub-Saharan Africa, including East, Central, West, and South Africa, for a number of reasons. For example, various cultures and languages exist within and between countries in the regions, which may have an impact on the (perceived) burden of dementia. In addition, previous reviews highlighted a number of limitations, including a lack of population-based studies, since most studies recruited participants from both patient care and population-based settings, and had an uneven representation of sub-Saharan Africa region.^22–24^ For instance, only four studies from East African countries were included in a previous review published in 2022.^10^ The projected increase in individuals living with dementia in the highly populous region of eastern sub-Saharan Africa emphasizes the need for improved characterization of the prevalence of dementia and cognitive impairment in this region. This is essential for several reasons. First, it provides a baseline for monitoring trends and projecting future healthcare needs in the region. Second, it informs policy development, allowing for the implementation of targeted interventions to improve diagnosis, treatment, and support services for affected individuals and their families. Third, it highlights the importance of research collaborations and capacity building initiatives to enhance local expertise in studying and addressing the complex factors contributing to dementia and cognitive impairment.

Therefore, the aim of this scoping review was to provide an overview of existing literature on the prevalence of dementia and cognitive impairment, and their associated factors based on population-based studies in East Africa, and to highlight research gaps that may inform future research studies and preventive strategies or initiatives in the region.

## MATERIALS AND METHODS

### Search strategy

We conducted a scoping review (rather than a systematic review) since we did not formulate a specific research question but aimed at providing an overview of available evidence, and highlighting research gaps. Literature search was done using three electronic databases (i.e., PubMed, Scopus, Google Scholar). The search terms included variations and combinations of pertinent keywords such as “dementia”, “cognitive impairment”, “prevalence”, and “East Africa”. Studies on the prevalence of dementia and/ or cognitive impairment conducted in East Africa and published prior to November 30, 2023 were considered in the review. We also screened reference lists of detected studies. Please refer to Supplementary Material 1 for more information on the search strategy.

### Inclusion and exclusion criteria

Inclusion criteria: 1) Original studies published in English on research conducted in East African countries, i.e., Burundi, Comoros, Djibouti, Ethiopia, Eritrea, Kenya, Rwanda, Seychelles, Somalia, South Sudan, Sudan, Tanzania, and Uganda. We did not include studies published in French or any other language spoken in East Africa since we assumed that findings from pertinent epidemiological studies would be published in English rather than French or any other (local) language, and manuscripts published in French or any other (local) language likely have an English abstract which we would have detected through our search; 2) studies reporting the prevalence of dementia and/ or cognitive impairment; 3) studies conducted in community/ population-based settings; and 4) studies conducted among participants aged≥50 years. We excluded conference abstracts, commentaries, (systematic) reviews and letters to the editors.

### Screening, selection, and data extraction

We used EndNote x9 software to merge search results from different databases and remove duplicates. Screening of publications by titles, abstracts and finally full texts was conducted independently by two authors (MAY and BFG). Any disagreement was resolved by discussion. A data extraction sheet was prepared and approved in consultation with the senior author (YEG). The data extraction sheet included information on first author, publication year, study setting, study design, sample size, prevalence of dementia and/ or cognitive impairment, and potential factors associated with dementia or cognitive impairment.

### Data synthesis

We synthesized data and information from included studies based on the following themes: 1) prevalence of dementia (including information on the diagnostic tool/ assessment); 2) prevalence of cognitive impairment (including information on the diagnostic tool/ assessment); 3) determinants of dementia and cognitive impairment; and 4) knowledge gaps in the East Africa context.

### Reporting

This scoping review followed the Preferred Reporting Items for Systematic Review and Meta-Analyses Extension for Scoping Reviews (PRISMA-ScR) checklist and reporting guideline^26^ to ensure transparent and comprehensive reporting of the review process. The extracted data, including prevalence rates and associated risk factors, are summarized and presented in tables and text.

## RESULTS

### Characteristics of included studies

**Fig. 1:**
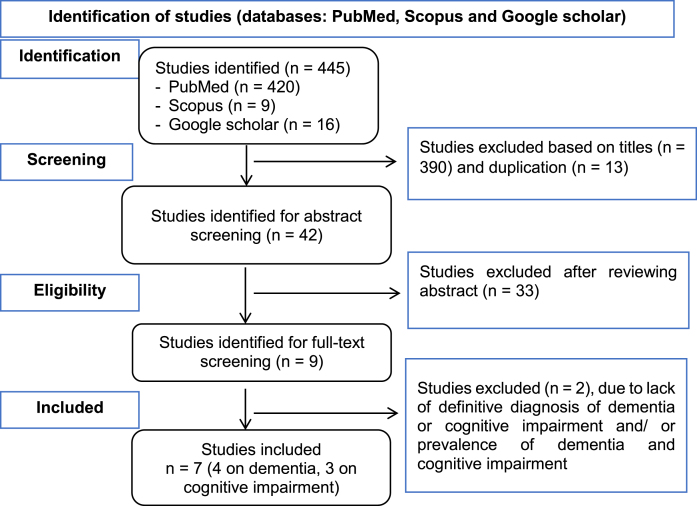
Flow chart of the selection process for the studies included in the scoping review.

The search yielded a total of 445 studies (420 from PubMed, 9 from Scopus, and 16 from Google Scholar). Thirteen duplicate studies were removed. After title, abstract, and full-text screening, 7 studies were finally included in the review (please refer to Fig. 1 for a flow chart of the screening process). Three studies were from Tanzania,^27 - 29^two from Uganda,^30,31^ and two from Ethiopia.^32,33^ With regard to study design, two were follow-up (longitudinal) studies, and the remaining were cross-sectional studies.

Four studies reported the prevalence of dementia,^28–31^and three reported the prevalence of cognitive impairment.^16,19,27^ The age of study participants varied across studies, e.g., participants aged≥70 years were included in a study to determine the prevalence of dementia in Tanzania, while participants aged≥50 years were included in studies to determine the prevalence of dementia and cognitive impairment in Uganda and Ethiopia, respectively (Table 1).

**Table 1:** Reports of prevalence and risk factors of dementia and cognitive impairment in East Africa from population-based studies

**Studies on dementia**							
Authors, y	Settings	Sample size	Population	Screening tool	Diagnostic criteria	Prevalence	Risk factors
Longdon, 2013^29^	Tanzania, rural	1,198	≥70 y, 56.2% female	CSI-D	DSM-IV; 10/66 Dementia research group	6.4% (DSM-IV); 21.6% (10/66 Dementia Research Group)	Old age: aOR (95% CI) 4.0 (1.85–8.78)
Yoseph, 2021^28^	Tanzania, rural	3,011	≥60 y, 57.3% female	IDEA six-item screen and IDEA IADL (brief version)	DSM-V	6.1% (4.6% age-adjusted) in those aged≥60 y 10.2% (8.6% age-adjusted) in those aged≥70 y	Old age: aOR (95% CI) 5.06 (1.68–15.26)
Mubangazi, 2020^30^	Uganda, rural	400	≥60 y, 59.5% female	Brief CSI-D		19.5%	Old age: aOR (95% CI) 1.02 (1.01–1.03); formal education: aOR (95% CI) 0.68 (0.49–0.96); physical inactivity: aOR (95% CI) 2.27 (1.39–3.70); lack of a ventilated kitchen: aOR (95% CI) 1.35 (1.30–4.17)
Benyumiza, 2023^31^	Uganda	434	≥50 y, 65.9% female	CSID		23.0% (95% CI, 19.2–27.3)	Old age: aOR (95% CI) 2.5 (1.5–4.1); positive history of nervous system infections (cerebral malaria: aOR (95% CI) 5.6 (2.6–12.0) and Herpes Simplex Virus-I: aOR (95% CI) 2.1 (1.1–4.1)); chronic headache: aOR (95% CI) 1.73 (1.04–2.87)
**Studies on cognitive impairment**							
Authors, y	Settings	Sample size	Population	Screening tool	Diagnostic criteria	Prevalence	Risk factors
Fekadu, 2022^32^	Ethiopia, Urban	423	≥60 y, 38.8% female	MMSE		42.1% (95% CI, 37.5–46.7)	Having no spouse: aOR (95% CI) 1.76 (1.08–2.86); depression: aOR (95% CI) 3.04 (1.80–5.14); lifetime alcohol use: aOR (95% CI) 2.90 (1.19–7.07); having low family support: aOR (95% CI) 3.07(1.35–6.96)
Gela, 2022^33^	Ethiopia, Urban	393	≥50 y, 42.5% female	MMSE		43.8% (95% CI, 38.8–48.7)	Old age: aOR (95% CI) 7.03 (2.78–17.77); low literacy (inability to read and write): aOR (95% CI) 5.05 (2.04–12.50); low-income level: aOR (95% CI) 2.60 (1.26–5.20); female sex: aOR (95% CI) 2.52 (1.50–4.26); poor social support: aOR (95% CI) 2.5 (1.30–4.81); rural residence: aOR (95% CI) 2.39 (1.26–4.51)
Paddick, 2015^27^	Tanzania, rural	296	≥70 y	CSI-D		7.0% (6.3% age-adjusted prevalence of MCI)	

CSI-D, Cognitive Screening Instrument for Dementia; DSM-IV, Diagnostic and Statistical Manual of Mental Disorders IV; IDEA, Identification and Intervention for Dementia in Elderly Africans; IADL, instrumental activities of daily living; MMSE, Mini-Mental Status Exam; MCI, mild cognitive impairment; aOR, adjusted Odds Ratio; 95% CI, 95% confidence interval.

### Prevalence and determinants of dementia

Two studies from Tanzania reported low population-based prevalence rates of dementia (6.1% and 6.4%), and two studies from Uganda reported slightly higher prevalence rates (19.5% and 23.0%). In the same study population using different diagnostic criteria for dementia in Tanzania, low (6.4%) and high (21%) prevalence of dementia was reported using Diagnostic and Statistical Manual of Mental Disorders IV (DSM-IV) and the 10/66 Dementia Research Group criteria, respectively. With regard to determinants examined in the studies, older age (adjusted Odds Ratio, aOR: 1.02–5.06), physical inactivity (aOR: 2.27; 95% confidence interval, CI, 1.39–3.70), and lack of a ventilated kitchen (aOR: 1.35; 95% CI, 1.30–4.17) were associated with increased odds of having dementia; whereas, formal education was associated with decreased odds of having dementia (aOR: 0.68; 95% CI, 0.49–0.96). In addition, having a history of central nervous system infections (i.e., cerebral malaria (aOR: 5.6; 95% CI, 2.6–12.0) and Herpes Simplex Virus-I (aOR: 2.1; 95% CI, 1.1-4.1)) and chronic headaches (aOR: 1.73; 95% CI, 1.04–2.87) were also reported as determinants of dementia (Table 1).

### Prevalence and determinants of cognitive impairment

Two studies from Ethiopia reported high prevalence rates of cognitive impairment (42.1% and 43.8%), and one study from Tanzania reproved low prevalence of cognitive impairment (6.3%). With regard to determinants examined in the studies, older age (aOR: 7.03; 95% CI, 2.78–17.77), female sex (aOR: 2.52; 95% CI, 1.50–4.26), a lower level of literacy (aOR: 5.05; 95% CI, 2.04–12.50), having depression (aOR: 3.04; 95% CI, 1.80–5.14), having no spouse (aOR: 1.76; 95% CI, 1.08–2.86), increased lifetime alcohol consumption (aOR: 2.90; 95% CI, 1.19–7.07), and having low family support (aOR: 2.5–3.07) were associated with increased odds of having cognitive impairment. Furthermore, having low income (aOR: 2.60; 95% CI, 1.26–5.20) and rural residence (aOR: 2.39; 95% CI, 1.26–4.51) were also documented as determinants of cognitive impairment (Table 1).

## DISCUSSION

Our review shows that the prevalence of dementia in East Africa based on four population-based studies ranges from 6.1% to 23%.^29–31,34^ Similarly, inconsistencies on prevalence estimates of dementia in population-based studies were also reported for other regions of Africa.^10,23^ The prevalence rates in our review are up to two times higher compared to prevalence (11.0%) reported in Southern Africa.^35^Relatively lower prevalence rates of dementia have also been reported in Western Africa, i.e., Benin (2.6% –3.7%)^36,37^ and Nigeria (4.9%),^38^and in Central Africa Republic (7.6%–8.1%).^39,40^ The prevalence of dementia is also reported to vary in Eastern Mediterranean countries, e.g., 3.8% and 14.3% in Egypt, and 12.2% in Morocco.^41^ These geographical variations in the prevalence estimates of dementia could be due to different diagnostic criteria used, and/ or methodology employed,^19^ including also linguistic differences in how symptoms are described in native languages. Furthermore, differences in prevalence rates might also be attributed to potentially modifiable risk factors that may vary between regions, such as proportion of education level, physical activity and social support.^9,42^

In addition to dementia, our review also includes three studies on the population-based prevalence of cognitive impairment in East Africa, with rates ranging from 7% to 44%. Interestingly, the prevalence of cognitive impairment in Tanzania (6.3%) among community-dwelling persons aged≥70 years was considerably lower than in Ethiopia (43%) among persons aged≥50 years. This large variation is in part attributable to misclassification bias as the study in Ethiopia used only Mini-Mental-Status Examination, a screening instrument which was not designed to diagnose cognitive impairment. Similar to dementia, inconsistent findings on the prevalence of cognitive impairment in population-based studies were also reported in other regions of Africa, e.g., Benin (10.2%),^36^ Congo (28.5%),^19^ the Central African Republic (37.9%),^39^ Nigeria (20.9%),^43^ Cameroon (33.3%),^17^ and South Africa (16.9%).^44^ Variations and differences in prevalence estimates across studies might be due to different diagnostic tools used to assess cognitive impairment, lack of established criteria for cognitive impairment (like they exist for dementia), and socio-demographic and economic differences between participants from different studies. For example, a community screening interview was used for studies in Central Africa Republic and Republic of Congo, and the Mini-Mental-Status Examination was used in Ethiopia. In addition, the age of included participants ranged considerably between studies. For example, individuals aged≥50 years were included in the studies conducted in Ethiopia^32^ and Cameroon,^17^while individuals aged≥60 years were included in another study in Ethiopia,^33^ and individuals aged≥65 years were included in the study in Benin.^36^

In our review, old age (with aOR ranging between 1.02 and 7.03) is a common, non-modifiable risk factor of dementia^30,31,34^ and cognitive impairment,^33^ which is consistent with prior research.^10,35,44^ Potential mechanisms underlying the association between higher age and increased risk of cognitive impairment and dementia have been discussed in the literature, including but not limited to a decrease in neurotransmitters, gray matter volume, and neocortical synapses as a result of aging.^45,46^ Cerebrovascular reactivity is also impaired with increasing age, which may result in brain hypoperfusion,^47^and the risk of having multimorbidity increases, which may in turn increase the risk of cognitive impairment and dementia.^9,48^

In addition, female sex was associated with increased odds of cognitive impairment, which is also consistent with previous studies.^9,10,17–19^ Observed sex differences in cognitive impairment and decline may be due to a variety of factors such as socioeconomic (e.g., literacy, income and and longevity), lifestyle, geographic, and environmental factors.^18^ Cognitive changes and complaints are also more common during menopause due to changes in sex hormone levels which have an impact on brain health and cognitive function.^49^ In addition, age at menopause and presence of hormone replacement therapy are associated with incidence of vascular cognitive impairment.^50^ In our review, high rates of illiteracy along with low income was common among females. On the other hand, females tend to live longer than males which may result in higher prevalence of cardiovascular diseases,^9^ and may impact the risk of cognitive impairment.

In the studies conducted in Tanzania and Uganda, low educational attainment was associated with increased odds of having dementia, and low formal education was also one modifiable risk factor for cognitive impairment in this review. Conflicting findings on the association between education and dementia have been reported in the literature,^29,35,51,52^ which may be due to differences in informal lifestyle choices shaped by culture that are thought to be protective against cognitive decline.

Physical inactivity, lack of a ventilated kitchen, and history of central nervous system infection and chronic headaches were also associated with increased odds of dementia in our review. Previous studies reported inconsistent findings on the association between these factors and dementia,^53–58^and more research is thus required. In addition, human immunodeficiency virus may be another important factor to consider in future studies, since rates of infection and access to treatment are relevant to dementia prevalence in East Africa, and should thus be considered in a holistic assessment of dementia prevalence rates. Finally, in this review, presence of depression, having no spouse, increased lifetime alcohol consumption, low income, rural residence and low family support were associated with increased odds of cognitive impairment, which has also been reported by otherstudies.^14,42,59–62^

Epigenetic alterations have been associated with dementia and cognitive impairment, especially at the level of DNA methylation, and such changes may help explain the observed interindividual variability in the development of these pathologies.^63,64^ Studies on the design of epigenetic biomarkers for disease detection, and for developing therapeutic strategies to improve cognitive impairment and dementia course are important.^64^ However, except for a convenience sample study that reported an APOE candidate gene in East Africa,^65^to the best of our knowledge, there is no population-based genetics or epigenetics research on dementia and cognitive impairment in East Africa. Indeed, such studies are needed to address the public health challenges of dementia and cognitive impairment.

Of note, only four studies on the prevalence of dementia, and three studies on the prevalence of cognitive impairment conducted in East Africa were included in this review. In addition, data has come from only three countries (i.e., Tanzania, Uganda, Ethiopia) out of 13 countries in the region. Thus, the conclusions derived from our review may not be regarded as representative for the entire East Africa region, and may need to be revised as more research on the prevalence of dementia and cognitive impairment conducted in East Africa becomes available in the future. The sparsity of studies might be due to lack of experts in related fields (i.e., neurologists, gerontologists and neuropsychologists),^66,67^ and less priority given to cognitive impairment and dementia by healthcare stakeholders in East African countries. As stated in the introduction, no nation in the sub-Saharan Africa region, which includes East Africa, has a stand-alone or integrated national dementia strategy to direct efforts to enhance dementia prevention or care nationally.^9,10,68^

This review therefore highlights the pressing need of research by employing rigorous methodology, including but not limited to the design and conduct of well-designed, population-based, observational studies such as cross-sectional and prospective cohort studies, and the use of standardized and validated diagnostic criteria including cognitive assessment, preferably by a neuropsychologist fluent in the native language, to establish a more accurate understanding of the prevalence and ultimately, the burden of dementia and cognitive impairment in the East Africa region. Population-based studies play a crucial role in providing reliable estimates of the prevalence of these conditions within a defined population, and findings may inform public health policies, healthcare planning, and resource allocation. Such studies also allow for comparisons between different regions and populations, contributing to a comprehensive understanding of dementia and cognitive impairment, in Africa and globally. Finally, population-based studies are resource intensive and require expertise from researchers with regard to their design and conduct. Collaborative efforts among researchers, healthcare institutions, governmental bodies in East Africa, and international investigators and research institutions may thus be vital for successfully conducting population-based studies on dementia and cognitive impairment in East Africa. Such collaborations may facilitate access to resources, expertise, and funding necessary for conducting large-scale studies. Engaging local communities and stakeholders throughout the research process is critically important to enhance the relevance, acceptability, and impact of the studies, ultimately leading to improved healthcare outcomes.

Furthermore, using advanced methods such as neuroimaging, biomarker ascertainment and genetics are also vital for identifying potential risk or protective factors associated with cognitive impairment and dementia, and for devising prevention and therapeutic strategies. In addition, the overall burden of dementia with regard to economic cost or social impact is also under-researched in East Africa. Longitudinal studies that follow individuals over time to understand the progression of dementia and its impact on individuals, families, caregivers, and communities are thus recommended.

In conclusion, only few population-based studies on the prevalence and potential determinants of dementia and cognitive impairment exist in East Africa, with prevalence rates ranging considerably across countries. Non-modifiable (i.e., age, female sex) and modifiable factors (i.e., lower level of education, chronic diseases, depression, physical inactivity, lack of a ventilated kitchen, history of central nervous system infection, chronic headaches, having no spouse, increased lifetime alcohol consumption, low family support) were documented as determinants of dementia and cognitive impairment in these studies. Governments, international organizations, and research institutions should allocate funding for population-based studies on dementia and cognitive impairment in East Africa. Establishing collaborative research networks in Africa, and partnering with international investigators and research institutions may enhance the capacity and expertise for conducting population-based studies on dementia and cognitive impairment. Longitudinal studies on dementia and cognitive impairment may provide valuable insights on incidence rates and risk factors of dementia and cognitive impairment, and may inform the development of targeted interventions including preventive strategies.

## AUTHOR CONTRIBUTIONS

Muluken A. Yenesew (Conceptualization; Data curation; Investigation; Methodology; Visualization; Writing – original draft); Janina Krell-Roesch (Writing – review & editing); Betelhem Fekadu (Conceptualization; Data curation; Investigation; Methodology; Supervision; Writing – review & editing); Dabere Nigatu (Data curation; Writing – review & editing); Aklilu Endalamaw (Data curation; Investigation; Writing – review & editing); Alemtsehay Mekonnen (Writing – review & editing); Mulugeta Biyadgie (Writing – review & editing); Gizachew Y. Wubetu (Writing – review & editing); Alemu T. Debiso (Writing – review & editing); Kassu M. Beyene (Writing – review & editing); Teshome S. Kelkile (Writing – review & editing); Daniel A. Enquobahrie (Writing – review & editing); Tesfaye B. Mersha (Writing – review & editing); Danielle E. Eagan (Writing – review & editing); Yonas E. Geda (Conceptualization; Investigation; Methodology; Supervision; Writing – review & editing).

## Supplementary Material

Supplementary Material

## Data Availability

The studies included in this review are available from databases PubMed, Scopus, and Google Scholar.
