# Supplementary material for: Prevalence of Dementia and Cognitive Impairment in East Africa Region: A Scoping Review of Population-Based Studies and Call for Further Research
Source: J Alzheimers Dis. 2024 Aug 13;100(4):1121–31. doi: 10.3233/JAD-240381 (PMC11380225; doi:10.3233/JAD-240381)
Supplement: Supplementary Material [file jad-100-jad240381-s001.pdf]

Supplementary Material

Prevalence of Dementia and Cognitive Impairment in East Africa Region: A Scoping Review of Population-Based Studies and Call for Further Research

Supplementary Material 1. Search strategy

Key concepts	Synonyms	Boolean operators
Dementia	'Dysfunction, Cognitive' OR 'Mild Cognitive Disorders' OR 'Cognitive Dysfunction'	OR
Cognitive impairment	'Mild Cognitive Impairment'	OR
Prevalence	'Period Prevalences' OR ‘Point prevalences’ OR "Epidemiology"	AND
East Africa	((Burundi) OR (Comoros) OR (Djibouti) OR (Ethiopia) OR (Eritrea) OR (Kenya) OR (Rwanda) OR (Seychelles) OR (Somalia) OR (South Sudan) OR (Sudan) OR (Tanzania) OR (Uganda))	

Final search term (for PubMed)

(((("Cognitive Dysfunction"[Mesh]) OR (((((((((((((((((((((((((((('Cognitive Decline'[Title/Abstract]) OR ('Cognitive Declines'[Title/Abstract])) OR ('Cognitive Disorder'[Title/Abstract])) OR ('Cognitive Disorders'[Title/Abstract])) OR ('Mild Cognitive Disorders'[Title/Abstract])) OR (Mild Cognitive Disorder[Title/Abstract])) OR ('Cognitive Dysfunction'[Title/Abstract])) OR ('Cognitive Dysfunctions'[Title/Abstract])) OR ('Cognitive Impairment'[Title/Abstract])) OR ('Cognitive Impairments'[Title/Abstract])) OR ('Mild Cognitive Impairment'[Title/Abstract])) OR ('Mild Cognitive Impairments'[Title/Abstract])) OR ('Mental Deterioration'[Title/Abstract])) OR ('Mental Deteriorations'[Title/Abstract])) OR ('Dysfunction, Cognitive'[Title/Abstract])) OR ('Dysfunctions, Cognitive'[Title/Abstract])) OR ('Impairment, Cognitive'[Title/Abstract])) OR ('Impairments, Cognitive'[Title/Abstract])) OR ('Disorder, Cognitive'[Title/Abstract])) OR ('Disorders, Cognitive'[Title/Abstract])) OR ('Cognitive Impairment, Mild'[Title/Abstract])) OR ('Cognitive Impairments, Mild'[Title/Abstract])) OR ('Impairment, Mild Cognitive'[Title/Abstract])) OR ('Impairments, Mild Cognitive'[Title/Abstract])) OR ('Decline, Cognitive'[Title/Abstract])) OR ('Declines, Cognitive'[Title/Abstract])) OR ('Deterioration, Mental'[Title/Abstract])) OR ('Deteriorations, Mental'[Title/Abstract]))) AND ((("Prevalence"[Mesh]) OR (((((((('prevalence'[Title/Abstract]) OR ('Prevalences'[Title/Abstract])) OR ('Period Prevalence'[Title/Abstract])) OR ('Period Prevalences'[Title/Abstract])) OR ('Prevalence, Period'[Title/Abstract])) OR ('Point Prevalence'[Title/Abstract])) OR ('Point Prevalences'[Title/Abstract])) OR ('Prevalence, Point'[Title/Abstract]))) OR (("Epidemiology"[Mesh]) OR (((((((((((((((('Epidemiology'[Title/Abstract]) OR ('Epidemiologies'[Title/Abstract])) OR ('Epidemiologic Studies'[Title/Abstract])) OR ('Epidemiological Studies'[Title/Abstract])) OR ('Epidemiological Study'[Title/Abstract])) OR ('Epidemiologic Study'[Title/Abstract])) OR ('Studies, Epidemiological'[Title/Abstract])) OR ('Study, Epidemiological'[Title/Abstract])) OR ('Studies, Epidemiologic'[Title/Abstract])) OR ('Study, Epidemiologic'[Title/Abstract])) OR ('Social Epidemiology'[Title/Abstract])) OR ('Social Epidemiologies'[Title/Abstract])) OR ('Epidemiologies, Social'[Title/Abstract])) OR ('Epidemiology, Social'[Title/Abstract])) OR ('Epidemiologic'[Title/Abstract])) OR ('Epidemiological'[Title/Abstract]))))) OR (Dementia)) AND ((Burundi) OR (Comoros) OR (Djibouti) OR (Ethiopia) OR (Eritrea) OR (Kenya) OR (Rwanda) OR (Seychelles) OR (Somalia) OR (South Sudan) OR (Sudan) OR (Tanzania) OR (Uganda))
